# Comparative Analysis of the Stability and Performance of Double-, Triple-, and Quadruple-Cation Perovskite Solar Cells for Rooftop and Indoor Applications

**DOI:** 10.3390/molecules29122758

**Published:** 2024-06-10

**Authors:** Shahriyar Safat Dipta, Ashraful Hossain Howlader, Walia Binte Tarique, Ashraf Uddin

**Affiliations:** School of Photovoltaic and Renewable Energy Engineering, University of New South Wales, Sydney 2052, Australia; s.dipta@unsw.edu.au (S.S.D.); a.howlader@unsw.edu.au (A.H.H.); w.binte_tarique@unsw.edu.au (W.B.T.)

**Keywords:** perovskite solar cells, stability, chemical composition, degradation

## Abstract

The solar energy market is predicted to be shared between Si solar cells and third-generation photovoltaics in the future. Perovskite solar cells (PSCs) show the greatest potential to capture a share there as a single junction or in tandem with silicon. Researchers worldwide are looking to optimize the composition of the perovskite film to achieve an optimal bandgap, performance, and stability. Traditional perovskites have a mixture of formamidinium and methyl ammonium as the A-site cation in their ABX_3_ structure. However, in recent times, the use of cesium and rubidium has become popular for making highly efficient PSCs. A thorough analysis of the performance and stability of double-, triple-, and quadruple-cation PSCs under different environmental conditions was performed in this study. The performance of the device and the films was analyzed by electrical measurements (J–V, dark J–V, EQE), scanning electron microscopy, atomic force microscopy, photoluminescence, and X-ray diffraction. The quadruple-cation device with the formula Cs_0.07_Rb_0.03_FA_0.77_MA_0.13_PbI_2.8_Br_0.2_ showed the highest power conversion efficiency (PCE) of 21.7%. However, this device had the least stability under all conditions. The triple-cation device with the formula Cs_0.1_FA_0.6_MA_0.3_PbI_2.8_Br_0.2_, with a slightly lower PCE (21.2%), was considerably more stable, resulting in about 30% more energy harvested than that using the other two devices during their life cycle.

## 1. Introduction

The growth of human civilization demands more energy usage, whereas the reserve of traditional energy sources is constantly depleting [1,2]. As a result, the installation of new energy harvesting facilities is swiftly moving toward renewable sources. Solar energy, in particular, is the prominent form of renewable energy that will dominate the future energy market [3,4,5]. Though about 90% of solar energy comes from silicon-based solar cells, perovskite solar cells (PSCs) show the potential to make a market breakthrough soon, with both single-junction and tandem devices [4,6,7]. Being in an early stage of development compared to silicon PV, PSCs have already gained huge popularity for research worldwide. PSCs have seen the steepest incline in the power conversion efficiency (PCE), which increased from 3.8% to more than 26% in both p-i-n and n-i-p devices in less than two decades [8,9,10]. PSCs are suitable for most of the energy demands, including rooftop, commercial, greenhouse, and indoor applications [11,12,13]. Perovskite materials have several suitable properties for PV applications, including direct bandgap tunability, a high absorption coefficient, a long carrier lifetime, and shallow defects [14,15,16]. In addition, the solution processibility of all the films in PSCs enables a low-temperature, low-cost, roll-to-roll facile fabrication scheme [17,18,19,20]. Though the efficiency of PSCs has reached an acceptable level for commercial use, their current stability is much lower than that of other PV technologies. PSCs suffer from both intrinsic and extrinsic degradations during their operation under ambient conditions [21,22]. Moreover, even under controlled storage conditions, these cells degrade due to their poor intrinsic stability. Even with traditional and novel encapsulations, their degradation cannot be brought down to the required standards [23].

To stabilize the devices and make them more efficient, numerous studies have been undertaken around the world. The improvement of the internal stability of the perovskite film needs an optimum Goldschmidt tolerance factor (Equation (1)) and octahedral factor (Equation (2)) [24,25,26]. Using a larger cation at site A can increase the tolerance factor. Similarly, using a larger cation at the B site decreases the tolerance factor, while increasing the octahedral factor. Changing the composition of the perovskite is one of the methods to obtain better-performing and stable PSCs [27,28,29,30]. Changing the ratio of the A-site cations or introducing a new A-site cation does not change the bandgap or absorbance of the device, in particular. However, it can add or remove surface defects and create smoother surfaces with larger grains that lead to better performance [31,32,33]. In addition, the Goldschmidt tolerance factor and octahedral factor are changed once there is a change in any ratio. Therefore, the intrinsic stability is changed with the addition or modification of cations (and anions) in the perovskite formula. Traditionally, formamidinium (FA) and methyl ammonium (MA) have been used in different ratios for perovskite films. Recently, small amounts of cesium (Cs) and rubidium (Rb) have been used for obtaining devices with a higher PCE [34,35].

(1) $$t=\frac{r_{A}+r_{X}}{\sqrt{2}\left(r_{B}+r_{X}\right)}$$

(2) $$µ=\frac{r_{B}}{r_{X}}$$

Gao et al. used a quadruple-cation A-site perovskite to achieve a 22.8% PCE, while their control showed only a 17.2% PCE [36,37]. Saliba et al. incorporated Cs to make triple-cation PSCs with a 21.8% PCE [38]. In addition, several other recent studies have used triple-cation devices and achieved a high PCE [39,40,41]. The composition of the overall perovskite formula is different in different studies. In particular, the halide composition differs in most of them, which can change the device bandgap, stability factors, and PV performance. Moreover, using different halide ratios can also impact the absorption of the device, which impacts all other PV parameters of the device. Therefore, a thorough comparison of the performance and stability of double-, triple-, and quadruple-cation PSCs with popularly used compositions, keeping the halide composition unchanged, is missing in the literature.

Here, we used FA_0.6_MA_0.4_PbI_2.8_Br_0.2_, Cs_0.1_FA_0.5_MA_0.4_PbI_2.8_Br_0.2_, and Cs_0.07_Rb_0.03_FA_0.5_MA_0.4_PbI_2.8_Br_0.2_ as double-cation, triple-cation, and quadruple-cation perovskite films, respectively. The surface quality of the films was measured by scanning electron microscopy (SEM). Dark I–V analysis was performed to show the electrical characteristics of the devices under dark conditions, which is related to the defects of the perovskite film. For photovoltaic (PV) performance analysis, current–voltage (I–V) and external quantum efficiency (EQE) measurements were performed. Finally, the degradation and stability of the devices were measured at different temperatures, in storage environments, and under operational conditions. The 4-cation perovskite devices showed the highest PCE, while being the least stable one. The 3-cation perovskite devices were slightly less efficient than their 4-cation counterparts and the most stable ones. This study analyzed the performance and stability of PSCs based on the number of cations used at the A site of the perovskite structure, keeping the halide composition the same, a direct comparison that is missing in the literature. In addition, this study analyzed stability under various conditions, from storage to operating conditions at high temperatures. The results will help researchers understand the effect of adding more cations at the A site of the perovskite on the PCE and stability under different conditions.

## 2. Results and Analysis

**Figure 1:**
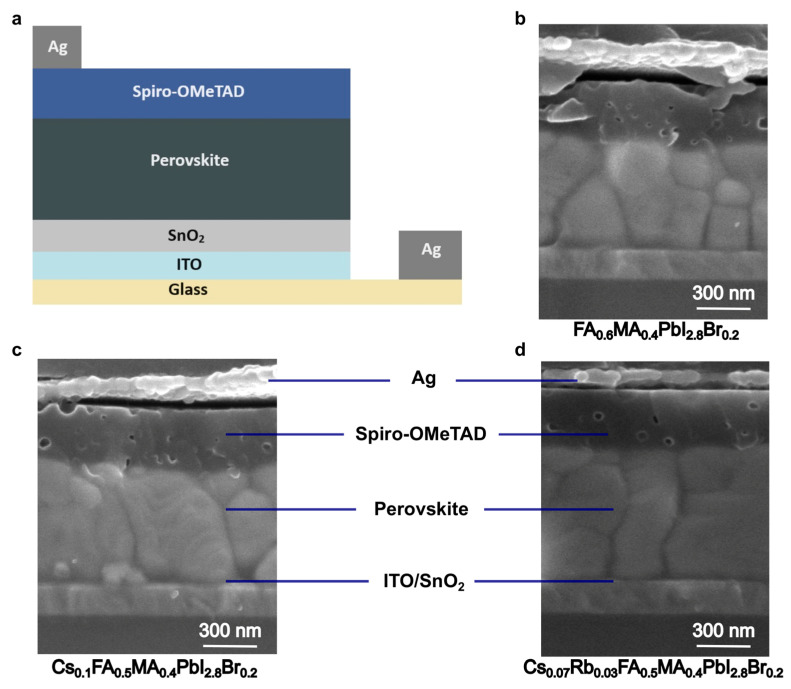
Structure of the full devices. (**a**) Schematic diagram. (**b**–**d**) Cross-section SEM images of the 2-cation, 3-cation, and 4-cation PSC devices.

The overall device had the structure glass/ITO/SnO_2_/perovskite/Spiro-OMeTAD/Ag, as shown in Figure 1a. Three different perovskite films were based on 2-cation-, 3-cation-, and 4-cation-based perovskites, as demonstrated by their cross-section SEM in Figure 1b–d.

**Figure 2:**
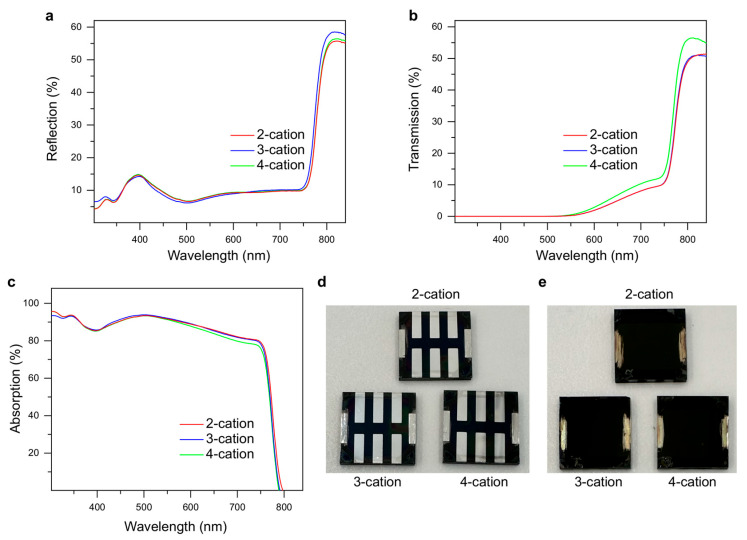
Optical properties of the devices. (**a**) Reflection, (**b**) transmission, and (**c**) absorption of the devices at different wavelengths of interest. (**d**) Back and (**e**) front side images of freshly prepared PSC samples. Each sample is a square of 144 mm^2^ area and contains six independent cells with common anodes.

Since the perovskite formula was changed in this study for the three devices, the optical reflection, transmission, and absorption could change. However, the halide composition in perovskite was kept the same such that the variation in the device optics was minimal. We performed optical measurements, as shown in Figure 2a–c, for the devices. As per the absorption analysis, the bandgap of the devices did not change with the formula. This is mostly because the halide composition was kept the same in all devices. Moreover, the reflection was only about 10% in the visible spectrum, which is quite low for a PSC without the use of an anti-reflective coating. The real images of the back and front sides of the devices are shown in Figure 2d and Figure 2e, respectively.

**Figure 3:**
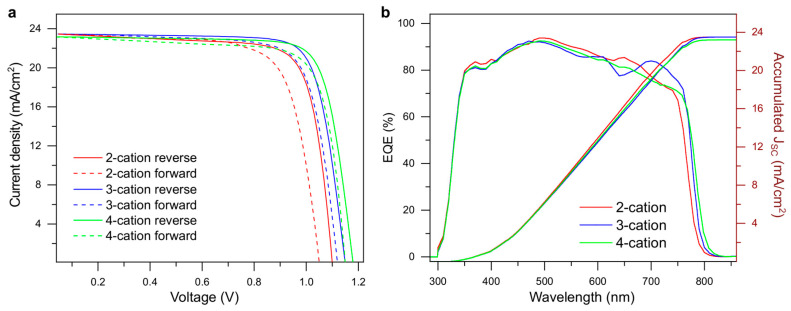
PV performance analysis of the 2-cation, 3-cation, and 4-cation PSCs. (**a**) The I–V plots of the cells in both forward and reverse directions to show the effect of hysteresis. (**b**) The EQE spectra of the devices under illumination conditions with a bias light.

**Figure A1:**
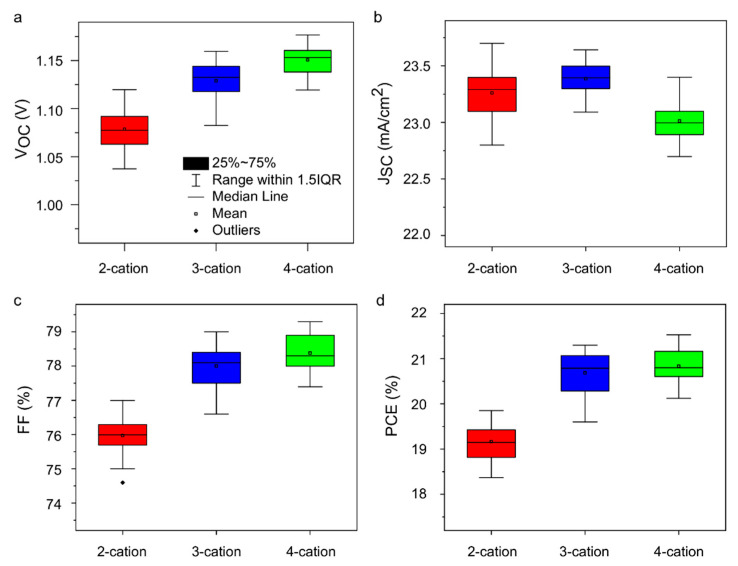
Statistical distribution of the I–V parameters (**a**) V_OC_, (**b**) J_SC_, (**c**) FF, and (**d**) PCE of 2-cation, 3-cation, and 4-cation PSCs used in this study.

The performance analysis of the devices for PV applications was carried out with I–V and EQE measurements, as shown in Figure 3a,b. The open-circuit voltage (V_OC_) increased from the 2-cation devices as Cs and Rb were added. However, the short circuit current density (J_SC_) showed a slight decline in the process. The fill factor (FF) of the cells also increased with the addition of more cations. Overall, the 4-cation devices showed the highest PCE, with 21.7% for the most efficient cell. The most efficient cell in the 3-cation devices was a close competitor, with a 20.7% PCE. Results of the statistical analysis of the I–V data for 20 cells of each type are shown in Figure A1. The PCE of the 3-cation devices was spread out more than that of the other two. However, they still maintained a higher average PCE value. The EQE of the devices showed almost similar spectra for all three devices, while the accumulated J_SC_ showed less than a 2% mismatch with J_SC_, found from I–V analysis. The PV parameters from the I–V and EQE analysis are presented in Table 1.

**Figure 4:**
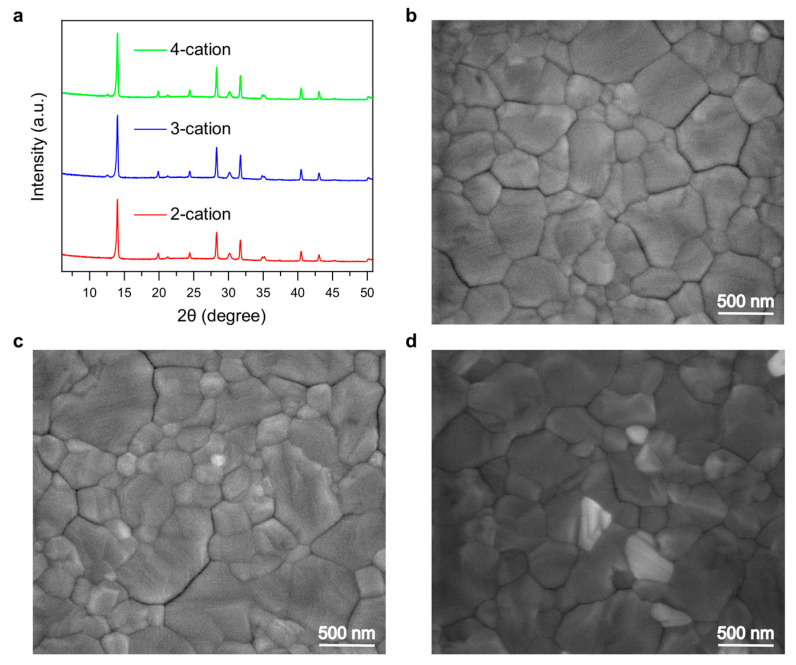
(**a**) XRD spectra of different types of perovskite films used in this study. (**b**–**d**) SEM images of the 2-cation (**b**), 3-cation (**c**), and 4-cation (**d**) perovskite films showing visible grains and boundaries.

**Figure A2:**
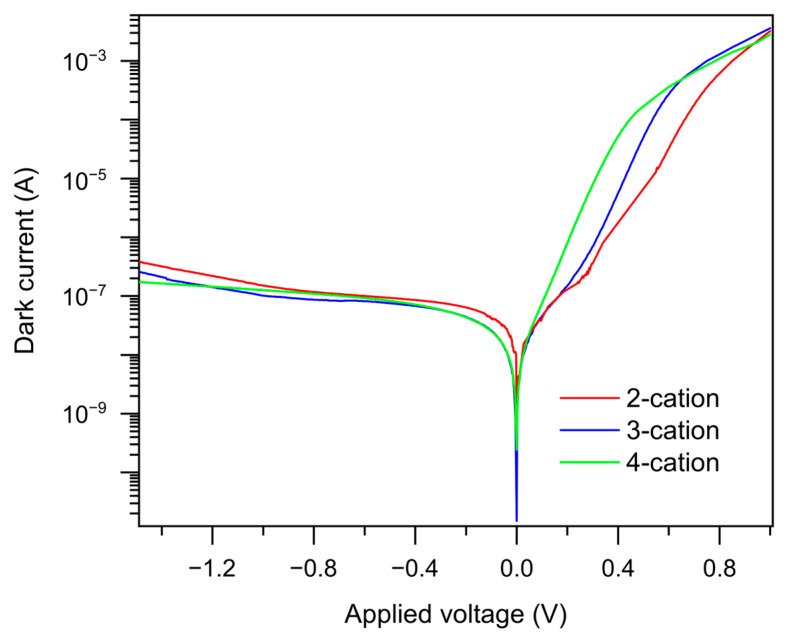
Dark I–V characteristics of different types of PSCs used in this study.

The electrical characteristics of the devices under dark conditions were obtained with dark I–V measurements, as shown in Figure A2. The leakage current (in reverse bias) was the lowest for 4-cation devices, whereas the 2-cation devices had the highest leakage current. This shows that bulk defects were reduced with the incorporation of Cs as the extra A-site cation in these devices. XRD was performed to analyze different materials present in the perovskite film. The perovskite characteristic peaks were found at 13.8 and 27.5 degrees (Figure 4a). The peaks were almost similar, and no visible differences were found in the overall XRD spectra, demonstrating that the perovskite films have similar X-ray responses. To further analyze the thin-film surface morphology, SEM of all three types of perovskite films was performed, as shown in Figure 4b–d. The grains of the 2-cation perovskite were found to be the most consistent. However, the grain size of the 3-cation perovskite was larger than that of its 2-cation counterpart. In addition, the 4-cation perovskite had even larger grains, as shown in Figure 4d, which resulted in better PV performance with fewer boundaries.

**Figure 5:**
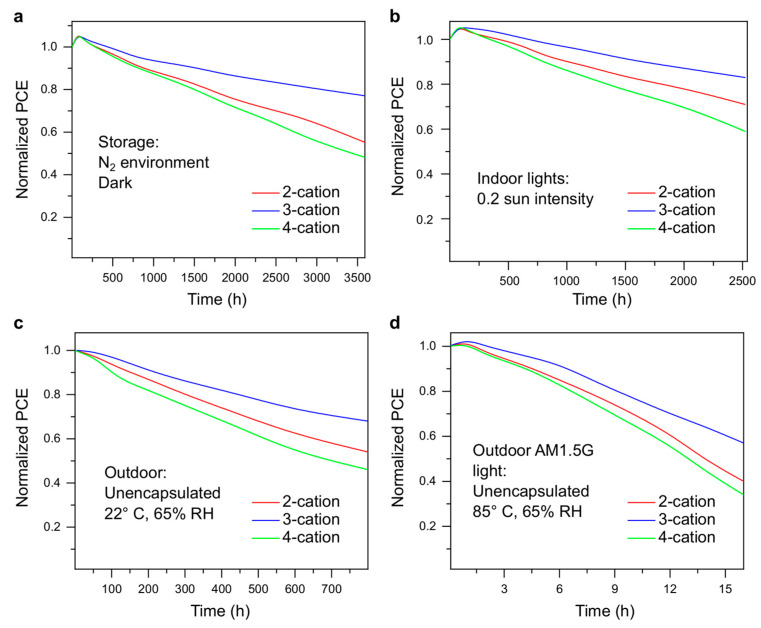
Stability analysis of different PSCs used in this study under (**a**) storage conditions, (**b**) indoor applications, (**c**) outdoor conditions, and (**d**) outdoor operating conditions.

Stability analysis is a crucial part of any perovskite device. Device stability was measured under various conditions in this study. Moreover, we performed stability analysis under low-sunlight (0.2 suns) conditions to demonstrate the long-term performance of the devices for indoor applications. The storage stability of the devices is shown in Figure 5a. Under storage conditions, all the degradation that occurred in the device was due to intrinsic degradation. The 3-cation devices outperformed the other two considerably. Figure 5b shows the stability of the devices over 110 days under indoor lights. The devices showed similar stability under storage conditions and under low-light conditions. Therefore, indoor applications suit PSCs more due to their degradation under outdoor conditions and illumination. To illustrate outdoor stability, the cells were kept outside under ambient conditions (22 °C and 65% RH), as shown in Figure 5c. The devices were faster to degrade, and the PCE of the most stable device (3-cation) dropped below 70% of the peak PCE mark in about 43 days. Finally, the devices were kept in AM1.5G sunlight, at 85 °C, and under 65% RH conditions for measuring the operational stability. The cells were fast to degrade, and the PCE of all the devices dropped below 60% of the initial PCE in ~15 h.

Though the stability in general was poor under both outdoor conditions (Figure 5c,d), note that these tests were carried out on unencapsulated PSCs. Encapsulating the cells can greatly improve their stability under outdoor conditions, in many cases close to the storage conditions if kept in the dark. In all the stability tests performed, the 3-cation devices were the most stable, while the 4-cation devices showed the fastest degradation. This shows that despite having the highest PCE initially, 4-cation devices are faster to degrade. On the contrary, a close competitor, 3-cation devices have a good PCE, and they are much better in terms of stability.

The XRD patterns of the perovskite films were analyzed before and after each of the stability tests. It was observed that the perovskite characteristic peaks at ~13.5 and ~27 degrees were decreased with aging. The relative decrease in the peak intensity was much more severe in the 4-cation perovskite film than in the other two. However, the surface morphology was also changed with aging, which added several pinholes and more grain boundaries. However, from the surface morphology, no conclusive evidence of additional degradation of one film compared to another was observed.

The ionic radius of Cs^+^ (181 pm) is slightly lower than that of FA and MA ions (180–210 pm). Therefore, adding 10% of Cs in the perovskite formula decreases the tolerance factor (Equation (1)) from 0.833 to 0.829. However, the value is still within the stable limit (0.8–1.0) for perovskites. Being an inorganic ion, Cs^+^ can form stronger chemical bonds, resulting in improved stability of perovskite films. On the contrary, adding a smaller Rb^+^ ion (140 pm) creates a strain on the crystal structure of the perovskite, allowing it to deform with much lower energy. As a result, the stability of 4-cation PSCs decreases with the addition of Rb. From the integrated area of the stability plots, the energy generated from 3-cation devices should be around 30% more than that generated from 4-cation devices under similar conditions. As a result, 3-cation devices should be preferred for long-term usage, even though they have a slightly lower starting PCE.

## 3. Discussion

Due to the flexibility of the modification of the perovskite formula, huge numbers of different perovskites have been used in recent years to yield a higher PCE. In general, adding more cations has often resulted in an improved PCE and fill factor. However, the Goldschmidt tolerance factor and octahedral factor are changed by adding different ions to or changing the ratio of different ions in the perovskite formula. This study showed the effect of adding one and two additional cations (Cs and Rb) to the already used FA and MA in the perovskite formula. The resulting devices showed that the initial PCE is the highest when all four cations used have the formula Cs_0.07_Rb_0.03_FA_0.5_MA_0.4_PbI_2.8_Br_0.2_. They are also similar optically, which shows that the increased PV performance is not due to more photon absorption. It was observed that the average grain size was increased with the addition of more cations to the A site. As a result, grain boundaries were reduced, along with trap sites for electrons and holes. Due to the decrease in defects and traps, the V_OC_ and the FF increased with the incorporation of more cations, as evidenced by their I–V plots.

However, detailed stability testing revealed that the 4-cation devices were the least stable intrinsically. In addition, they were degraded faster than both the control (2-cation) and the 3-cation devices under all environmental conditions. Crystallization of the perovskite material plays a huge role in the stability of the devices. Adding more cations distorts the crystals, and they tend to break down more easily into their precursors. The difference in the ionic radii of the cations also puts a strain on the crystals, leading them to degrade faster. The most stable 3-cation devices, which are just 0.6% (absolute) less efficient than their 4-cation counterparts, can produce around 30% more energy in their lifetime under similar conditions. Therefore, they are the most suitable perovskite for use in solar cells, keeping long-term energy harvesting as the primary purpose.

## 4. Materials and Methods

### 4.1. Materials

ITO substrates and Spiro-OMeTAD were purchased from Luminescence Technology Corp. (Lumtec Corp), Zhubei, Taiwan. Formamidinium iodide (FAI), methylammonium iodide (MAI), and methylammonium bromide (MABr) were purchased from GreatCell Solar Materials (Queanbeyan, Australia). Lead iodide (PbI_2_) and lead bromide (PbBr_2_) were purchased from TCI (Portland, OR, USA). All solvents, cesium iodide (CsI), and rubidium iodide (RbI) were purchased from Sigma Aldrich (St. Louis, MA, USA) and used as received. Tin oxide (SnO_2_) dispersion solution (15% in H_2_O) was bought from Thermo Fisher Scientific Limited (St. Bend, OR, USA). Silver shots were bought from ESPI Metals (Ashland, OR, USA).

### 4.2. Device Fabrication

Patterned ITO on glass substrates (12 mm × 12 mm) was cleaned sequentially with Hellmanex III, deionized water, acetone, and 2-propanol in an ultrasonic bath, with 15 min spent in each step. The cleaned substrates were then heated at 70 °C for 30 min and UV–ozone-treated for a further 20 min. For the electron transport layer, 15% SnO_2_ dispersion solution was mixed with distilled water in a ratio of 1:3 to make it 3.75% and then stirred and spin-coated at 3000 rpm for 35 s on clean ITO substrates. The sample was transferred to an open-air hot plate and annealed for 30 min at 150 °C to form the mesoporous SnO_2_ layer.

For double-cation perovskite: One molar FA_0.6_MA_0.4_PbI_2.8_Br_0.2_ solution was prepared by mixing 103.2 mg of FAI, 63.6 mg of MAI, 419.9 mg of PbI_2_, and 36.7 mg of PbBr_2_ and dissolving the mixture in 1 mL solution of DMF and DMSO in a ratio of 4:1.

For triple-cation perovskite: One molar Cs_0.1_FA_0.5_MA_0.4_PbI_2.8_Br_0.2_ solution was prepared by mixing 86 mg of FAI, 63.6 mg of MAI, 26 mg of CsI, 419.9 mg of PbI_2_, and 36.7 mg of PbBr_2_ and dissolving the mixture in 1 mL solution of DMF and DMSO in a ratio of 4:1.

For quadruple-cation perovskite: One molar Cs_0.07_Rb_0.03_FA_0.5_MA_0.4_PbI_2.8_Br_0.2_ solution was prepared by mixing solutions from three bottles. In the first bottle (B1), 135.1 mg of FAI, 22.7 mg of MABr, 395.14 mg of PbI_2_, and 40.4 mg of PbBr_2_ were mixed and dissolved in a 1 mL solution of DMF and DMSO in a ratio of 4:1. In the second bottle (B2), 120.7 mg of CsI was dissolved in 500 mL of DMSO. In the third bottle (B3), 98.6 mg of RbI was dissolved in 500 mL of DMSO. All three bottles were stirred rigorously for 2 h. Next, 70 mL of the solution from B2 and 30 mL of the solution from B3 were added to B1, which was the final solution for the perovskite film deposition. The final solution in B1 was stirred for a further 2 h.

For the perovskite film deposition, the solution was spin-coated at 1000 rpm for 10 s, followed by 4000 rpm for 40 s. The perovskite film was immediately annealed at 110 °C for 10 min.

For the hole transport layer (HTL), 72.3 mg of Spiro-OMeTAD solution was dissolved in 1 mL of chlorobenzene. To increase the conductivity, 28.8 μL of 4-tBP and 17.5 μL of Li-TFSI salt were added and the mixture rigorously stirred for 2 h. The solution was then spin-coated on the perovskite thin film at a rate of 3500 rpm for 30 s.

Finally, the electrodes of the device were thermally evaporated using a shadow mask at a rate of 0.1 nm/s under 10^−5^ m-bar pressure. The electrodes were 100 nm thick. For the first 10 nm, the rate was maintained at 0.01 nm/s. The cells had an active area of 0.045 cm^2^ each.

### 4.3. Film and Device Characterization

The current versus voltage (I–V) data were measured in an I–V testing system equipped with a Keithley 2400 source meter from PV Measurements Inc. (Stratford, CT, USA). The source light was equipped with an AM1.5G filter and calibrated with a reference silicon cell from PV Measurements Inc. to adjust for the desired sun intensity. External quantum efficiency (EQE) measurements were carried out with a QEX7 Spectral Response System from PV Measurements, Inc. (Boulder, CO, USA). Absorbance, reflectance, and transmittance measurements were performed on a UV–VIS–NIR spectrometer (PerkinElmer–Lambda 950, Seer Green, Beaconsfield, UK). The surface topology and cross-section images were characterized by NanoSEM 450 (scanning electron microscopy) fitted with a retractable annular backscattered electron detector, as well as a Bruker SDD-EDS detector (Cramlington, UK). X-ray diffraction (XRD) characterization was performed on a PANalytical Empyrean Thin-Film XRD machine (Almelo, The Netherlands) with CuKα radiation by step-scanning with a step size of 0.02°. Dark I–V measurements were performed on an Autolab PGSTAT-30 analyzer (Utrecht, The Netherlands).

## Figures and Tables

**Table 1:** Summary of the J–V performance of the most efficient device and the average (20 samples) of each type.

Device		V_OC_ (mV)	J_SC_ (mA/cm^2^)	FF (%)	PCE (%)	R_S_ (Ω-cm^2^)	R_Sh_ (Ω-cm^2^)
2-cation	Most efficient	1102	23.5	76.7	19.8	3.2	812
	Average	1084 ± 44	23.3 ± 0.5	76.0 ± 1.4	19.2 ± 0.8	3.8 ± 1.1	3051 ± 2239
3-cation	Most efficient	1147	23.5	78.8	21.3	3.9	2727
	Average	1134 ± 44	23.4 ± 0.3	78.0 ± 1.4	20.7 ± 1.1	4.6 ± 0.9	3672 ± 974
4-cation	Most efficient	1177	23.2	79.3	21.7	3.8	2250
	Average	1154 ± 35	23.0 ± 0.4	78.4 ± 1.0	20.9 ± 0.7	4.7 ± 0.8	2978 ± 957

## Data Availability

The data that support the findings of this study are available from the corresponding author upon reasonable request.

## Appendix A. Evaluation of Grain Size from Surface SEM

The diameter of an average-size grain was noted from five SEM images of different areas for each perovskite film. The grain size of the perovskite films was evaluated based on the average diameters.
